# Multiomics approach unravels fertility transition in a pigeonpea line for a two‐line hybrid system

**DOI:** 10.1002/tpg2.20028

**Published:** 2020-06-18

**Authors:** Lekha T. Pazhamala, Palak Chaturvedi, Prasad Bajaj, Sandhya Srikanth, Arindam Ghatak, Annapurna Chitikineni, Anke Bellaire, Anupama Hingane, C.V. Sameer Kumar, K.B. Saxena, Wolfram Weckwerth, Rachit K. Saxena, Rajeev K. Varshney

**Affiliations:** ^1^ Center of Excellence in Genomics & Systems Biology International Crops Research Institute for the Semi‐Arid Tropics (ICRISAT) Patancheru Hyderabad 502 324 India; ^2^ Molecular Systems Biology (MOSYS), Department of Functional and Evolutionary Ecology, Faculty of Life Sciences University of Vienna Althanstrasse 14 Vienna 1090 Austria; ^3^ Department of Botany and Biodiversity Research University of Vienna Rennweg 14 Vienna 1030 Austria; ^4^ Crop Improvement Theme International Crops Research Institute for the Semi‐Arid Tropics (ICRISAT) Patancheru Hyderabad 502 324 India; ^5^ Vienna Metabolomics Center (VIME) University of Vienna Vienna Austria; ^6^ Institute of Agriculture University of Western Australia 35 Stirling Highway Crawley WA 6009 Australia

## Abstract

Pigeonpea [*Cajanus cajan* (L.) Millsp.] is a pulse crop cultivated in the semi‐arid regions of Asia and Africa. It is a rich source of protein and capable of alleviating malnutrition, improving soil health and the livelihoods of small‐holder farmers. Hybrid breeding has provided remarkable improvements for pigeonpea productivity, but owing to a tedious and costly seed production system, an alternative two‐line hybrid technology is being explored. In this regard, an environment‐sensitive male sterile line has been characterized as a thermosensitive male sterile line in pigeonpea precisely responding to day temperature. The male sterile and fertile anthers from five developmental stages were studied by integrating transcriptomics, proteomics and metabolomics supported by precise phenotyping and scanning electron microscopic study. Spatio‐temporal analysis of anther transcriptome and proteome revealed 17 repressed DEGs/DEPs in sterile anthers that play a critical role in normal cell wall morphogenesis and tapetal cell development. The male fertility to sterility transition was mainly due to a perturbation in auxin homeostasis, leading to impaired cell wall modification and sugar transport. Limited nutrient utilization thus leads to microspore starvation in response to moderately elevated day temperature which could be restored with auxin‐treatment in the male sterile line. Our findings outline a molecular mechanism that underpins fertility transition responses thereby providing a process‐oriented two‐line hybrid breeding framework for pigeonpea.

## 1 INTRODUCTION

Feeding a world population which is expected to reach 9.8 billion by 2050 under natural resource scarcity and weather extremes will be challenging, especially in the semi‐arid tropics. Pigeonpea [*Cajanus cajan* (L.) Millsp.] is a resilient food legume crop capable of alleviating malnutrition, improving soil conditions and livelihood of the smallholder farmers, especially in Asia and Africa. It is a diploid (*2n = 2x = 22*), often cross‐pollinated crop with 833.07 Mb genome belonging to the Phaseoleae tribe (Varshney et al., 2012; Young, Mudge, & Ellis, 2003). The natural out‐crossing ability of pigeonpea has led to the establishment of a cytoplasmic‐genetic male sterile (CGMS) line‐based hybrid breeding technology (Saxena, Tikle, Kumar, Choudhary, & Bahadur, 2016). This short‐lived perennial crop is mainly adapted to tropical environment with an optimum temperature range of 25–35 °C for growth and development. Based on the maturity of the crop, it has been categorized as extra‐early, early, medium and late maturing having different photoperiod responses. The early‐maturing group being the least sensitive while the late‐maturing the most sensitive (Saxena, 2008). Despite varietal improvement, pigeonpea productivity has remained stagnant at around 700 kg ha^−1^ over last six decades. Hybrid breeding technology, however, has shown positive indications of genetic enhancement with 30–40% on‐farm yield advantage over popular varieties (Saxena et al., 2016; Saxena & Nadarajan, 2010).

A hybrid breeding technology based on CGMS system involves three different parental lines, namely male sterile (A‐), its maintainer (B‐) and restorer (R‐) lines. This three‐line hybrid seed production technology is not only cumbersome but also resource intensive. The B‐ and the R‐lines are fertile inbred lines which are multiplied in isolation plots, in addition to the seed production for hybrid (A × R) and A‐line (A × B) which is undertaken in separate isolated plots. This hybrid technology requires specific male sterile, maintainer and fertility restorer lines, and this restrict breeders from utilizing the valuable germplasm resources (Chen, Xiao, & Lei, 2010; Zhang et al., 2013). Alternatively, a two‐line hybrid breeding system would simplify the entire hybrid seed production by utilizing an environment‐sensitive genic male sterile (EGMS) line. Such lines exhibit fertility transition under specified environmental conditions such as temperature, photoperiod and radiation (Kaul, 1988). The term ‘fertility transition’ here means that the male sterile plants start producing viable pollen to become male fertile plant and vice‐versa when exposed to a set of different environmental conditions. Any elite line with better yield, grain quality and resistance to diseases and/or pests could be utilized to restore fertility (Chang et al., 2016; Fan et al., 2017). EGMS lines have been widely utilized in rice to develop high‐yielding two‐line based hybrids with up to 10% higher yield than the three‐line hybrid rice (Chang et al., 2016; Kim & Zhang, 2018; Pan et al., 2014). However, the successful application of this technology requires a well‐characterized EGMS line with defined locations/seasons for hybrid and self‐seed production. An unpredictable transition of the male sterility to fertility under field conditions could lead to a failure of the hybrid seed production. Utilization of a two‐line hybrid system and understanding of the genes involved in cytoplasmic male sterility (CMS), photoperiod‐sensitive genic male sterility (PGMS) and thermosensitive genic male sterility (TGMS) has made tremendous progress in rice. However, it is not the case in other cereals and legumes. Role of MYB transcription regulator in rice (Zhang et al., 2013), PHD‐finger protein in soybean (Thu et al., 2019), pectin‐degradation‐related genes in wheat (Li et al., 2019) and non‐coding RNAs in rice (Wu et al., 2019) have been reported in different crops. In the case of pigeonpea, some of the lines derived from a cross involving a wild relative (*Cajanus sericeus* Benth. Ex. Bak) and a cultivar (ICPA 85010) demonstrated marked changes in the fertility status of the plants under field conditions (Saxena, 2014). In this regard, identification and characterization of some of these stable lines for their utilization in a two‐line hybrid system was undertaken in the present study. Detecting the key environmental ‘cue’, critical developmental stage(s), genes involved and understanding the molecular mechanisms of fertility transition is critical for the success of a two‐line hybrid breeding system. To unravel developmental patterns and complex multigenic regulation of fertility transition, it is important to analyze and integrate all molecular levels (transcripts, proteins and metabolites) which reflect the complete information flow from the gene to metabolism to phenotype in the environmental context (Weckwerth, Ghatak, Bellaire, Chaturvedi, & Varshney, 2020). In the present study, we have performed precise phenotyping of some pigeonpea EGMS lines under highly controlled growth conditions. Together with cytological analysis and multiomics analyses including anther stage‐specific transcriptomics, proteomics and metabolomics, we propose a working model explaining the molecular mechanisms underlying fertility transition. The findings of this study would enable the development of an effective two‐line hybrid breeding system for rapid, inexpensive and high‐quality hybrid pigeonpea seed production.

## 2 MATERIALS AND METHODS

### 2.1 Plant materials and growth conditions

Pigeonpea male sterile lines developed as a result of a multiple cross genome transfer between *Cajanus sericeus* Benth. Ex. Bak., a wild relative of pigeonpea and an elite cultivar of *Cajanus cajan* (L.) Millsp. showing fertility transition were used in the present study (Ariyanayagam, Nageshwara, & Zaveri, 1995; Saxena et al., 2010). Eight of these environment‐sensitive male sterile lines, namely *Envs Sel 101–108* were selected to study at International Crops Research Institute for the Semi‐Arid Tropics (ICRISAT), Patancheru, India (17.53°N latitude, 78.27°E longitude). For evaluation of the select lines, pollen viability analysis was carried out to examine the sterility/fertility status of the plants of each line in response to various combinations of environmental factors. Anthers were crushed to stain the mature pollen grains with a 1% acetocarmine solution. Stained pollen grains were counted as fertile grains whereas partial to unstained grains were considered as sterile. Pollen sterility/fertility was evaluated by calculating the pollen sterility percentage (PSP). Alexander's stain (Alexander, 1969) which stains fertile pollen red and sterile pollen green, was also used to assess reliable percentages of pollen viability during critical transition stages using a bright field microscope (Olympus BH2 attached with Nikon E4500).

Three EGMS lines, namely *Envs Sel 104*, *Envs Sel 105* and *Envs Sel 107* were selected for evaluation under glasshouse conditions. For each line, a total of twelve plants were grown and maintained as three sets of four plant, which were evaluated for their fertility transition under glasshouse and growth chamber conditions. In the growth chamber, ramping up and ramping down of temperature and light intensity was set for 2 h for the day and night transition period to mimic dawn and twilight under natural conditions. To capture temperature variations precisely inside the growth chamber, three data loggers were fixed at three different levels (near the top, middle and the lower branches) of the plants. A set of four plants were used to study the response to varying day length, temperature, humidity and light intensities under growth chamber (Conviron, Winnipeg, Canada) conditions. The set was swapped with the other set of four plants to confirm the result after subjecting a condition for at least a period of 15 days on a completely new inflorescence. It was ensured that all old inflorescence was nipped off when subjecting to the new regime. Details of the environmental conditions used to study the key factors responsible for fertility transition have been summarized in Supplemental Table S1.

**Core Ideas**

- We identified a pigeonpea thermosensitive male sterile line, *Envs Sel 107*, which precisely responds to day temperature.
- A multiomics approach revealed an impairment of cell wall modification and sugar transport in the male sterile line.
- Exogenous application of auxin was found to reverse male sterility in *Envs Sel 107* line.
- We have proposed a molecular mechanism explaining male fertility transition in response to day temperature.

### 2.2 Microscopic examination of pollen

#### 2.2.1 Bright field microscopy

Characterization of microsporogenesis and pollen fertility was performed using microscopic examination. Immature flower buds were fixed in Carnoy's II solution (1:3:6 acetic acid–chloroform–ethanol) for 24 h and transferred to Carnoy's I solution (1:3 acetic acid–ethanol) for 72 h at 4 °C for cytological analysis of meiocytes. Meiocytes were squashed and stained in 4% acetocarmine and Pollen Mother Cells (PMCs) were examined and photographed using a bright field microscope (Olympus BH2 attached with Nikon E4500). Table 1 provides detail of the flower bud stage corresponding to the pollen development stages considered for this study.

**TABLE 1:** Details of the flower bud stages corresponding to the pollen development stages considered for different studies

Pollen development stage	Flower bud stages	Designation of stages	Sterile sample	Fertile sample	‐Omics data generated
Pollen mother cells	Stages: A, B, C	Stage 1	S1	F1	Anther transcriptome, Anther proteome
Tetrad	Stages: D, E, F	Stage 2	S2	F2	Anther transcriptome, Anther proteome, Anther metabolome
Microspore	Stages: G, H, I, J, K	Stage 3	S3	F3	Anther transcriptome, Anther proteome, Anther metabolome, SEM
Maturing pollen	Stages: L, M, N, O, P, Q	Stage 4	S4	F4	Anther transcriptome, Anther proteome, Anther metabolome, SEM
Desiccated pollen (Anthesis stage)	Stages: R, S, T	Stage 5	S5	F5	Anther transcriptome, Anther proteome

*Notes*: SEM, scanning electron microscopy

#### 2.2.2 Light microscopy (LM) analysis

Air dried pollen grains of Stage 3 and Stage 4 were observed in a drop of water on a slide under a light microscope (System Microscope BX 50; Olympus, London UK). The images were captured with a digital camera (DS‐Fil; Nikon, Tokyo, Japan).

#### 2.2.3 Scanning electron microscopy (SEM) analysis

Pollen wall was analyzed using air dried pollen of Stage 3 and Stage 4 which were first rehydrated in 70% ethanol and then dehydrated in a series of ethanol (85%, 96%) followed by acetone. After critical point drying using an Autosamdri‐815 (Tousimis, USA), the samples were mounted on a stub and sputter coated with gold using a sputter coater (SCD 050, USA). The images of the pollen grains were obtained in a scanning electron microscope (JSM‐6390, JEOL, USA).

### 2.3 Tissue harvesting for transcriptome, proteome and metabolome analyses

Anther samples were harvested on ice from three different *Envs Sel* 107 plants in 100% sterile condition and then converted to more than 98% fertile conditions under the identified conditions for sterile and the fertile samples, respectively. These three plants were considered as the three biological replicates. The buds were immediately sorted out according to their developmental stages on ice and stored in RNAlater (Sigma #R0901) at 4 °C until anther separation. Here, anther tissues were considered for this study to capture transcriptional changes in tapetum and developing pollen together. Moreover, the separation of sporogenous tissues and pollen was practically difficult, especially during Stage 1 which was very tiny and resulted in a dearth of samples to process while Stage 5 was almost a dried mass hard to separate. Anthers from the buds were carefully harvested removing all other vegetative tissues including filaments with the help of a stereo microscope in liquid nitrogen after confirming the developmental stages (Olympus, Tokyo, Japan), and stored at −80 °C until further use. Total RNA, protein and metabolites were extracted from three biological replicates separately and pooled in equimolar concentrations which represented the sample for transcriptomics, proteomics and metabolomics, respectively. In the case of transcriptomics, one technical replicate while for proteomics and metabolomics, three technical replicates were used.

### 2.4 Transcriptome sequencing and RNA‐Seq analysis

#### 2.4.1 Total RNA isolation and transcriptome sequencing

Total RNA was isolated from 10 to 20 mg anthers using the Ambion RNAqueous‐Micro kit (Ambion #AM1931) according to the manufacturer's instructions. The quantity and quality of RNA samples were estimated using Qubit RNA Assay Kit (Thermo Fisher Scientific Inc., USA) and Agilent RNA 6000 Nano chip on Agilent 2100 Bioanalyzer (Agilent Technologies, Palo Alto, CA, USA), respectively. The equimolar concentration of total RNA isolated from the three biological replicated was pooled and ∼2.5 µg of initial sample was used to isolate the mRNA. Illumina TruSeq RNA Sample Preparation v2 LS Kit (Illumina Inc., San Diego, CA) was utilized to prepare library of template molecules for subsequent cluster generation and sequencing using Illumina HiSeq 2500 platform at Center of Excellence in Genomics & Systems Biology at ICRISAT.

#### 2.4.2 RNA‐Seq data pre‐processing and analysis

Paired end reads of 125 bases generated using Illumina HiSeq 2500 platform were subjected to quality filtering using Trimmomatic v0.33 (Bolger, Lohse, & Usadel, 2014). The RNA‐seq data generated were analysed for global and differential gene expression using the “Tuxedo suite” (Trapnell et al., 2012). This includes TopHat v2.1.0 (Kim et al., 2013) and Bowtie v2.2.5 (Langmead & Salzberg, 2012) to align and map the reads of all the samples to the pigeonpea genome (http://www.icrisat.org/gt-bt/iipg). The mapped reads were then assembled into genes and isoforms using a reference annotation‐based transcript (RABT) approach with Cufflinks v2.2.1 (Roberts, Pimentel, Trapnell, & Pachter, 2011). Finally, DEGs were identified using cuffdiff (Trapnell et al., 2010) and their functional annotation was performed. Hierarchical clustering of differentially expressed genes was performed using MeV v4.8.1 (Saeed et al., 2003) with Pearson's correlation as similarity metrics.

#### 2.4.3 Functional annotation

All the assembled genes were subjected to BLASTX search against NCBI's non‐redundant (nr) Viridiplantae protein database. Gene Ontology (GO) annotation and pathways were assigned using Blast2GO v4.1 (Götz et al., 2008). The list of genes found to have expressed exclusive to a sample was generated by calculating Tissue specificity index (τ) using the equation:

$$\tau \;=\frac{\sum_{i=1}^{N}\left(1-x_{i}\right)}{N-1}$$

where N is the number of samples and x*_i_* is the expression value of a gene normalized by maximum value across all samples (Yanai et al., 2005). Thus, the specifically expressed genes were identified with τ = 1. Genes with similar expression pattern were identified by K‐means clustering using MeV v4.8.1 with Euclidean distance as similarity metrics.

### 2.5 Protein extraction and proteomic analysis

#### 2.5.1 Total protein extraction

Proteins were extracted and analysed from anthers corresponding to the five pollen developmental stages (Table 1) according to Chaturvedi, Ischebeck, Egelhofer, Lichtscheidl, and Weckwerth (2013); Chaturvedi et al. (2015); and Jegadeesan et al. (2018). Anther tissues were freeze‐dried and grinded for 2 min in a shaking mill using steel balls (2 mm diameter). The homogenized samples were suspended in 200 µl of protein extraction buffer (100 mM Tris‐HCl, pH 8.0; 5% SDS, 10% glycerol; 10 mM DTT; 1% plant protease inhibitor cocktail; Sigma P9599) and incubated at room temperature for 5 min followed by incubation for 2.5 min at 95 °C and centrifugation at 21000 *g* for 5 min at room temperature. The supernatant was carefully transferred to a new tube. Two‐hundred microliters of 1.4 M sucrose were added to the supernatant and proteins were extracted twice with 200 µl TE buffer‐equilibrated phenol followed by counter extraction with 400 µl of 0.7 M sucrose. Phenol phases were combined and subsequently mixed with 2.5 volumes of 0.1 M ammonium acetate in methanol for precipitation of proteins. After 16 h of incubation at −20 °C, samples were centrifuged for 5 min at 5000 *g*. The pellet was washed twice with 0.1 M ammonium acetate, once with acetone and air‐dried at room temperature. The pellet was re‐dissolved in 6 M Urea and 5% SDS and protein concentration were determined using the bicinchoninic acid assay (BCA method). Proteins were pre‐fractionated by SDS‐PAGE. Forty micrograms of total protein were loaded onto gel. Gels were fixed and stained with methanol/acetic acid/water/Coomassie Brilliant Blue R‐250 (40:10:50:0.001). Gels were destained in methanol/water (40:60).

#### 2.5.2 Protein digestion and LC‐MS/MS

Gel pieces were destained, equilibrated and digested with trypsin, desalted and concentrated (Chaturvedi et al., 2013). Prior to mass spectrometric measurement, the tryptic peptide pellets were dissolved in 4% (v/v) acetonitrile, 0.1% (v/v) formic acid. One µg of each sample (3 replicates for sterile and fertile samples) was loaded on a C18 reverse phase column (Thermo scientific, EASY‐Spray 500 mm, 2 µm particle size). Separation was achieved with a 90 min gradient from 98% solution A (0.1% formic acid) and 2% solution B (90% ACN and 0.1% formic acid) at 0 min to 40% solution B (90% ACN and 0.1% formic acid) at 90 min with a flow rate of 300 nl min^−1^. nESI‐MS/MS measurements were performed on Orbitrap Elite (Thermo Fisher Scientific, Bremen, Germany) with the following settings: Full scan range 350–1800 m/z resolution 120000 max. 20 MS2 scans (activation type CID), repeat count 1, repeat duration 30 s, exclusion list size 500, exclusion duration 30 s, charge state screening enabled with the rejection of unassigned and +1 charge states, minimum signal threshold 500.

#### 2.5.3 Peptide and protein identification

Raw data were searched with the SEQUEST algorithm present in Proteome Discoverer version 1.3 (Thermo, Germany) as described previously (Ghatak et al., 2016). We have used the following settings in Proteome Discoverer for data analysis which include: Peptide confidence: High, which is equivalent to 1% false discovery rate (FDR), and Xcorr of 2, 3, 4, 5, 6 for peptides of charge 2, 3, 4, 5, 6. The variable modifications were set to acetylation of N‐terminus and oxidation of methionine, with a mass tolerance of 10 ppm for parent ion and 0.8 Da for the fragment ion. The number of missed and/or non‐specific cleavages permitted was 2. There were no fixed modifications, as dynamic modifications were used.

The transcript sequences from the RNA‐Seq data generated for the present work were utilized for protein identification. Peptides were matched against these databases plus decoys, considering a significant hit when the peptide confidence was high. The identified proteins were quantitated based on total ion count followed by a NSAF normalization strategy (Paoletti et al., 2006):

$${\left(NSAF\right)}_{\kappa }={\left(PSM/L\right)}_{\kappa }/\sum_{i=1}^{N}{\left(PSM/L\right)}_{i}$$

where the total number spectra count for the matching peptides from protein *k* (PSM) was divided by the protein length (*L*), then divided by the sum of PSM/L for all *N* proteins.

### 2.6 Metabolite extraction and data analysis

#### 2.6.1 Metabolite extraction

Metabolite extraction was performed as described previously (Weckwerth, Wenzel, & Fiehn, 2004), with slight modifications. Anthers (∼10–50 mg) corresponding to the five pollen developmental stages (Table 1) were freeze‐dried and ground for 2 min in a shaking mill using steel balls (2 mm diameter). The 750 µl extraction solution (methanol/chloroform/water (MCW) = 2.5:1:0.5 v/v) pre‐chilled was added to the tissue in safe lock eppendorf tubes, and thoroughly mixed at 4 °C for 15 min to precipitate proteins and DNA/RNA and to disassociate metabolites from membrane and cell wall components. Further, the tubes were centrifuged at 20,000 *g* for 4 min at 4 °C, and the supernatant was transferred to a new eppendorf tube. The remaining pellet was washed once again with 250 µl MCW precooled, vortexed thoroughly at 4 °C for 15 min, and centrifuged at 20,000 *g* for 4 min at 4 °C. The supernatant was pooled with the previously collected MCW, 300 ml water were added, and the emulsion was mixed and finally centrifuged for 2 min at 20,000 *g* at 25 °C. The upper phase (methanol/water) and the lower phase (chloroform phase) were collected from each sample and transferred to new eppendorf tubes separately and lyophilized in a vacuum evaporator (SCANVAC Cool Safe 110‐4, Speed Vacuum concentrator, Labogene).

#### 2.6.2 Metabolite profiling

Metabolite analysis was performed according to Weckwerth et al. (2004) and Obermeyer, Fragner, Lang, and Weckwerth (2013). Lyophilized samples of the polar phase (upper phase, methanol/water) were dissolved in 20 µl methoxylamine hydrochloride in pyridine (40 mg ml^−1^) and incubated for 90 min at 30 °C in a thermoshaker. Eighty microliters of N‐methyl‐N‐(trimethylsilyl) trifluoroacetamide (MSTFA) (Macherey Nagel) was spiked in all the samples and incubated at 37 °C for 30 min in a thermoshaker. After the incubation, the samples were centrifuged for 2 min at 14000 *g* and then transferred in GC‐microvials with micro inserts and closed with crimp caps. Along with the samples 60 µl retention index marker solution of even alkanes from C10 to C40 in hexane (Sigma‐Aldrich) at a concentration of 50 mg L^−1^ was also prepared with MSTFA spiked. For gas chromatography‐mass spectrometry (GC‐MS) analyses of primary metabolites, a LECO Pegasus 4D GC × GC TOFMS instrument was used. Samples, alkanes and blanks were injected with a split/splitless injector at a constant temperature of 230 °C. Injection volume was 1 ml of derivatized sample, injection was performed at a split ratio of 1:25. Gas chromatographic separation was conducted on HP‐5MS column (30 m × 0.25 mm × 0.25 mm, Agilent Technologies) using helium as carrier gas at a flow rate of 1 ml min^−1^. Temperature gradient started at 70 °C isothermal for 1 min, followed by a heating ramp of 9 °C min^−1^ to 330 °C held for 7 min. Transfer line temperature was 250 °C, and ion source temperature was set to 200 °C. Mass spectra were acquired with an acquisition rate of 20 spectra s^−1^ at a mass range of mass‐to‐charge ratio 40 to 600 thomson using a detector voltage of 1,500 V and electron impact ionization of 70 eV.

#### 2.6.3 Metabolite data analysis

Data processing and analysis were performed using ChromaTof (Leco) software. Chromatograms of different samples were used to generate a reference peak list, and all other data files were processed against this reference list. Retention index markers were used to calculate retention indices of compounds and for chromatographic alignment. Deconvoluted mass spectra were matched against an in‐house mass spectral library, and this retention index was used for peak annotation too. Peak annotations, as well as peak integrations, were checked manually before exporting peak areas for relative quantification into a Microsoft Excel program. Areas of different trimethylsilyl derivatives of single metabolites were summed, and from methoxyamine products, only one peak was selected for further analyses. All hydrophilic metabolite amounts are given in arbitrary units corresponding to the peak areas of the chromatograms (Obermeyer et al., 2013). Heatmap was generated using COVAIN toolbox in Matlab and Box plots were constructed using R script (package ggplot2).

### 2.7 Studying auxin treated sterile plants

#### 2.7.1 Auxin treatment of the male sterile inflorescence

Auxin was sprayed on the male sterile inflorescence from bud initiation stage to microspore stage grown in three replications under controlled growth conditions favouring male sterility. Plants were sprayed with 10^−4^ M auxin solution (Indole‐3‐acetic acid; Merk) as described in Sakata, Yagihashi, and Atsushi (2010). Mock treatment with distilled water was carried out on the control plants. All the treatment was carried out at a fixed time in the morning hours to avoid diurnal variation effects. Buds from Stage 4 were collected before anthesis for pollen viability analysis using acetocarmine staining from the auxin‐treated and control plants. Pollen sterility percentage was calculated precisely to determine the effect of auxin treatment on pollen viability.

#### 2.7.2 Expression analysis using qRT‐PCR

Expression analysis of nine auxin‐related genes was performed using Real‐time quantitative polymerase chain reaction (qPCR) in Applied Biosystems 7500 Real Time PCR System with SYBR Green chemistry (Applied Biosystems, USA). The reactions were conducted with three biological replicates using actin gene as endogenous control. The relative expression of auxin‐treated anther samples was calculated with respect to the untreated sterile samples. The ΔCt value for each of the genes was calculated with respect to the housekeeping gene and was expressed in fold change (2‐ΔΔCt) value as described previously (Livak & Schmittgen, 2001). The sequences of the primers used for qPCR has been provided in Supplemental Table S2.

## 3 RESULTS

### 3.1 Screening and selection of pigeonpea EGMS lines

Eight environment‐sensitive male sterile (EGMS) lines namely *Envs Sel 101–108* that showed reversion to fertility were grown in the experimental fields during June, July, and August 2012. The pollen sterility percentage (PSP) was recorded subsequently at the onset of flowering to assess the proportion of sterile plants in each line. Plants were categorized as male sterile when at least five randomly collected flowers from different branches showed an average of ≥ 90% non‐viable pollen. PSP was recorded during October to November 2012, November to December 2012, and December 2012 to January 2013 for the EGMS lines grown in June, July, and August 2012, respectively. The variation in minimum and maximum temperatures from June 2012 to February 2013 has been depicted in Supplemental Figure S1a. In the lines planted in July 2012, there has been a marked fertility transition observed in plants during November (15.78–28.71 °C; 11:08 to 11:28 h day length) to December (13.67–29.85 °C; 11:04 to 11:08 h day length), especially in the line *Envs Sel 104*, *105 and 107* (Supplemental Figure S1b). It was observed that that the percentage of plants displaying ≥ 90% PSP reduced from 98 to 28% from November to December, respectively in *Envs Sel 107*. *Envs Sel 104*, *105 and 107* were further closely evaluated under controlled growth conditions (Supplemental Figure S1c and 1d). *Envs Sel 107* was found to have the potential to exhibit fertility transition from male sterility (>90%) to fertility and then revert back to complete male sterility (>90%) which is required for the success of a possible two‐line hybrid system (Supplemental Figure S1b, highlighted, c, d). Therefore, *Envs Sel 107* was selected to understand fertility transition and key environmental factor, critical developmental stages and molecular mechanisms for its utilization for a two‐line hybrid system in pigeonpea.

### 3.2 Floral phenotypes and pollen morphology in EGMS line

Male sterile flowers were characterized by relatively longer pistils that caused the stigma to protrude above the stamen unlike male fertile flowers where the stamens and stigma were almost at the same level (Supplemental Figure S2a and 2b). However, there were no marked differences in terms of the arrangement and size of petals and sepals. At the dehiscence stage, anthers of the male sterile flowers looked dark brown in color and failed to dehisce (Supplemental Figure S2a), contrary to the fertile anthers that showed anther dehiscence, releasing viable pollen grains (Supplemental Figure S2b). Prior to this stage, anthers of male sterile flowers looked translucent with non‐viable pollen grains (Supplementary Figure 2c), whereas anthers looked distended with bright yellow colored viable pollen grains in male fertile flowers (Supplemental Figure S2d). Upon staining with acetocarmine stain, sterile pollen grains looked colorless (Supplemental Figure S2e) while fertile pollen grains took up the pink color (Supplemental Figure S2f). Interestingly, the non‐viable unstained pollen grains remained as tetrads and could not separate into microspore (Supplemental Figure S2g), whereas the tetrads dissociated into microspores to develop into viable pollen in fertile anthers (Supplemental Figure S2h).

### 3.3 Key environmental factors for fertility transition

During December month, *Envs Sel 107* line produced viable pollen grains (PSP ≤ 20%) and became male fertile, whereas pollen viability was lost (PSP ≥ 90%) in the month of January under field conditions. December month had comparatively cooler temperatures (13.67 °C minimum and 29.85 °C maximum) with slightly shorter photoperiod of 11:04 to 11:08 h than January (minimum 15.02 °C and maximum 29.98 °C temperature and 11:05 to 11:20 h of photoperiod). This variation in temperature and photoperiod makes remarkable changes in the plants showing fertility transition in the field conditions. Twelve plants of *Envs Sel 107* line were evaluated under controlled growth chamber conditions with varying combinations of night temperature, day temperature, day length, light intensity and relative humidity. To identify the key environmental factor(s) affecting fertility transition, only one factor was altered at a time, keeping other factors constant for a minimum of 15 days before recording PSP. In all, we studied more than 20 different combinations of environmental factors that could influence fertility transition using controlled growth conditions (Supplemental Table S1). The outcome of these experiments has been summarized in the following sections.

#### 3.3.1 Fertility transition with varying photoperiod

At ICRISAT‐Patancheru, photoperiod varies from 11 h (Short Day‐SD) to 13 h (Long Day‐LD) throughout the year. These two photoperiod regimes (minimum and maximum day length) were thus selected for evaluating the response of *Envs Sel 107* line in two different conditions namely, SD and LD grown at 30 ± 1 °C in the same glasshouse. A set of nine *Envs Sel 107* plants designated as SD was allowed a photoperiod from 6 AM to 5 PM (11 h of photoperiod) by blocking the natural sunlight using dark curtains. On the other hand, a similar set of plants designated as LD was subjected to a photoperiod from 5 AM to 6 PM (13 h of photoperiod) including dawn and twilight as experienced by plants under natural conditions. No fertility transition was recorded in any of the plants of SD (11 h) and LD (13 h) sets as indicated by PSP which recorded 100% male sterility. This result was reconfirmed using a set of four plants for their fertility response under very short photoperiod (6 h photoperiod) under growth chamber conditions keeping other factors such as temperature (30 °C day), humidity (50:80% day/night) and light intensity (350 µM m^−2^ s^−1^) in the growth chambers constant. All the plants under study remained completely male sterile indicating that photoperiod does not have a direct influence on the fertility transition of the *Envs Sel 107* line.

#### 3.3.2 Fertility transition with varying temperature

Effect of low temperature (LT) with a short day (SD) conditions and high temperature (HT) with long day (LD) conditions on fertility transition of *Envs Sel 107* line was studied, respectively in two different chambers referred to as LT+SD and HT+LD. These would mimic natural field conditions prevailing during winter (November‐December) and summer (February‐March) at ICRISAT‐Patancheru. Four plants each were subjected to LT+SD regime with 24/12 °C day/night and 11 h photoperiod and HT+LD conditions with 30/16 °C day/night and 13 h photoperiod. Surprisingly, all the plants showed 100% male sterility under both conditions. Next, the night temperature of LT+SD chamber was reduced from 12 °C to 10 °C and further to 8 °C with a constant day temperature of 24 °C. None of the plants showed any reversal of male sterility under these varying night temperatures. However, plants responded to lower day temperatures and produced fertile pollen when exposed to 18 °C (PSP dropped from 100% to 35%) and 21 °C (PSP dropped from 100% to 50%) in the LT+SD and HT+LD chamber, respectively, with all the other conditions similar. The experiments demonstrated the role of day temperature on fertility transition.

#### 3.3.3 Critical temperature defines thermosensitive male sterile line

**FIGURE 1:**
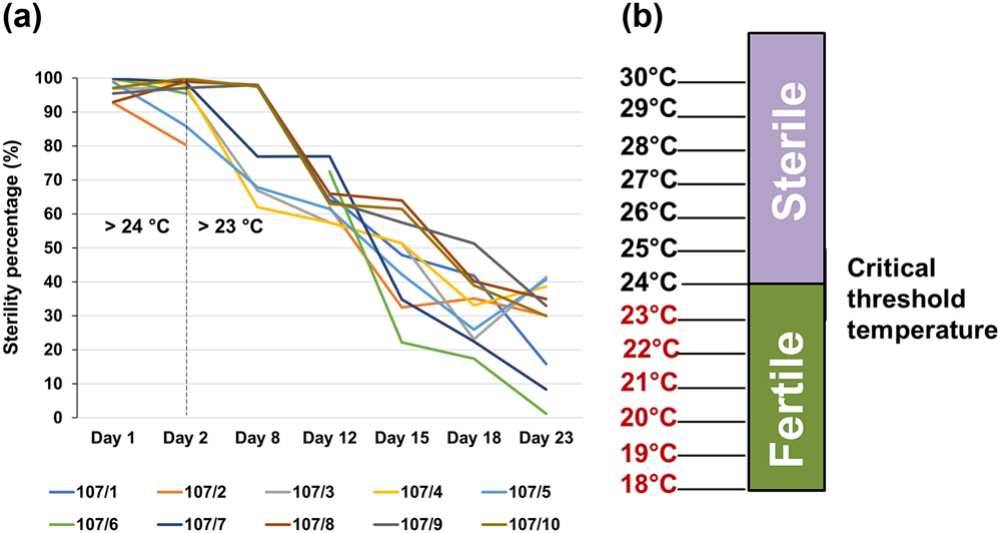
Identification of critical temperatures for fertility transition in *Envs Sel 107* line. (a) Pollen sterility percentage was monitored in ten different male sterile plants under controlled growth conditions. All the environmental factors were kept constant while the day temperature was gradually reduced (by 1 °C) from 26 °C to 23 °C until all the plants converted to male fertile. In two of the plants, there are some missing observations caused due to the non‐availability of flowers on that day. (b) A schematic depiction of critical temperatures identified for fertility transition. In the *Envs Sel 107* line, the critical (threshold) temperature responsible for reverting male sterility was determined after evaluating temperatures between 18 °C and 30 °C

Critical temperature (CT) or the threshold temperature for fertility transition in *Envs Sel 107* would be vital in identifying the locations and seasons for commercial hybrid seed production. In order to identify CT, day temperatures were evaluated using 10 completely male sterile plants namely ‘107/1’ to ‘107/10’. All the plants maintained at 30 °C with 100% PSP were monitored by reducing 1 °C temperature until it reached 18 °C keeping the night temperature (12 °C) and photoperiod (11 h) constant. Production of fertile pollen grains was recorded when the day temperature was reduced from 24 °C to 23 °C. The male sterile plants (PSP ≤ 100%) used for the study converted to male fertile (PSP ≤ 20%) over a period of 15–20 days (Figure 1). It was found that temperatures higher than 24 °C promoted male sterility while temperatures below 23 °C allowed fertile pollen grain formation. Thus, the day temperature of 24 °C is referred to as critical temperature as it is found to be the threshold temperature for fertility transition.

### 3.4 Post‐meiotic aberrations in pollen development causes sterility

**FIGURE 2:**
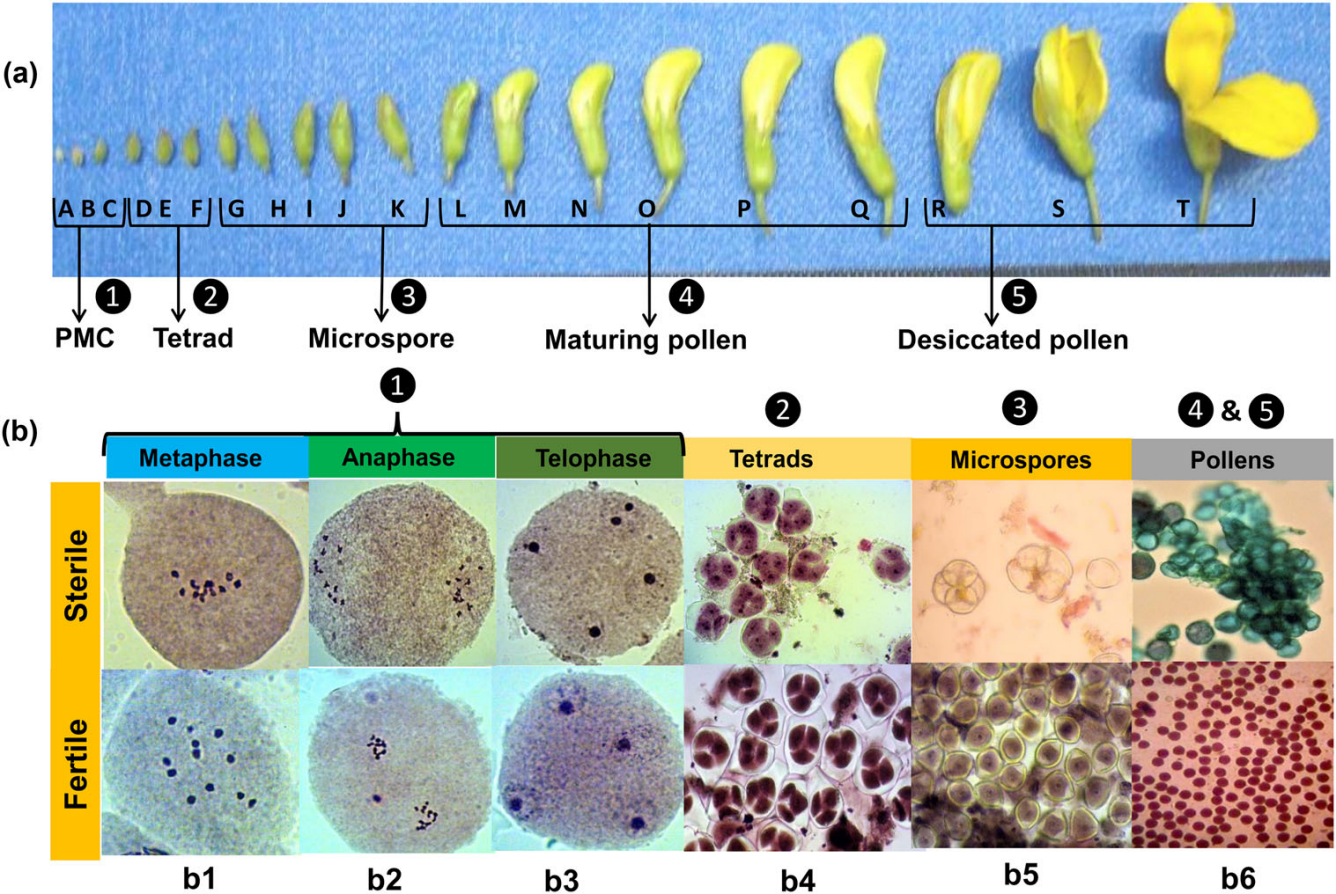
A comparative cytological study of male sterile and fertile anthers. (a) Identification of flower buds of 20 different sizes (A to T) that have been correlated to five stages of pollen development in pigeonpea. (b) Meiotic analysis in pollen mother cell and subsequent pollen developmental stages in sterile and fertile anthers. Figures b1, b2 and b3 show the photomicrograph of metaphase, anaphase and telophase, respectively. Figures b4, b5 and b6 present the photomicrograph of the tetrad, microspore and pollen development stages, respectively

A detailed study was conducted on sterile and fertile pollen development in *Envs Sel 107* line to identify the critical stage at which the pollen development seems to be disrupted in response to day temperature. Twenty different stages of flower development from bud initiation (Stage A) to opened flower (Stage T) were correlated to five major pollen development stages by conducting histological studies using stereomicroscope (Figure 2a; Table 1). Bud stages A to C corresponded to the pollen mother cell stage (Stage 1), bud stages D to F represented the tetrad stage (Stage 2), whereas bud stages G to K corresponded to microspore stage (Stage 3) of pollen development. Maturing pollen stage (Stage 4) were correlated to a broad bud stage ranging from L to Q which mainly represents the development of polarized microspore. Similarly, the desiccated or mature pollen grain stage corresponds to the Anthesis or the pollen release stage of flower, R to T. Distinguishing major stages was crucial for identifying the critical changes that lead to sterility when compared to fertile pollen development. The cells were viable until the tetrad stage, however, some meiotic abnormalities such as the formation of univalents at metaphase, chromosomal laggards at anaphase and dyads at the tetrad stage were observed in less than 3% of PMCs in the male sterile line.

However, the PMCs were found to have normal cytological divisions, subsequently forming tetrads in 97% observation. In brief, the male sterile line showed the development of normal PMCs when compared to the fertile counterparts until the tetrad stage (Figure 2b, 1 to 2b4). However, after the tetrad stage, we observed microspores that failed to separate, eventually losing viability as evident by the receding cytoplasm (Figure 2b, 5, 2b6). The possible reason for the male sterility seems to be the post‐meiotic defects in pollen development in response to an external environmental factor such as day temperature in this case.

### 3.5 Spatio‐temporal dynamics of anther transcriptome

**FIGURE 3:**
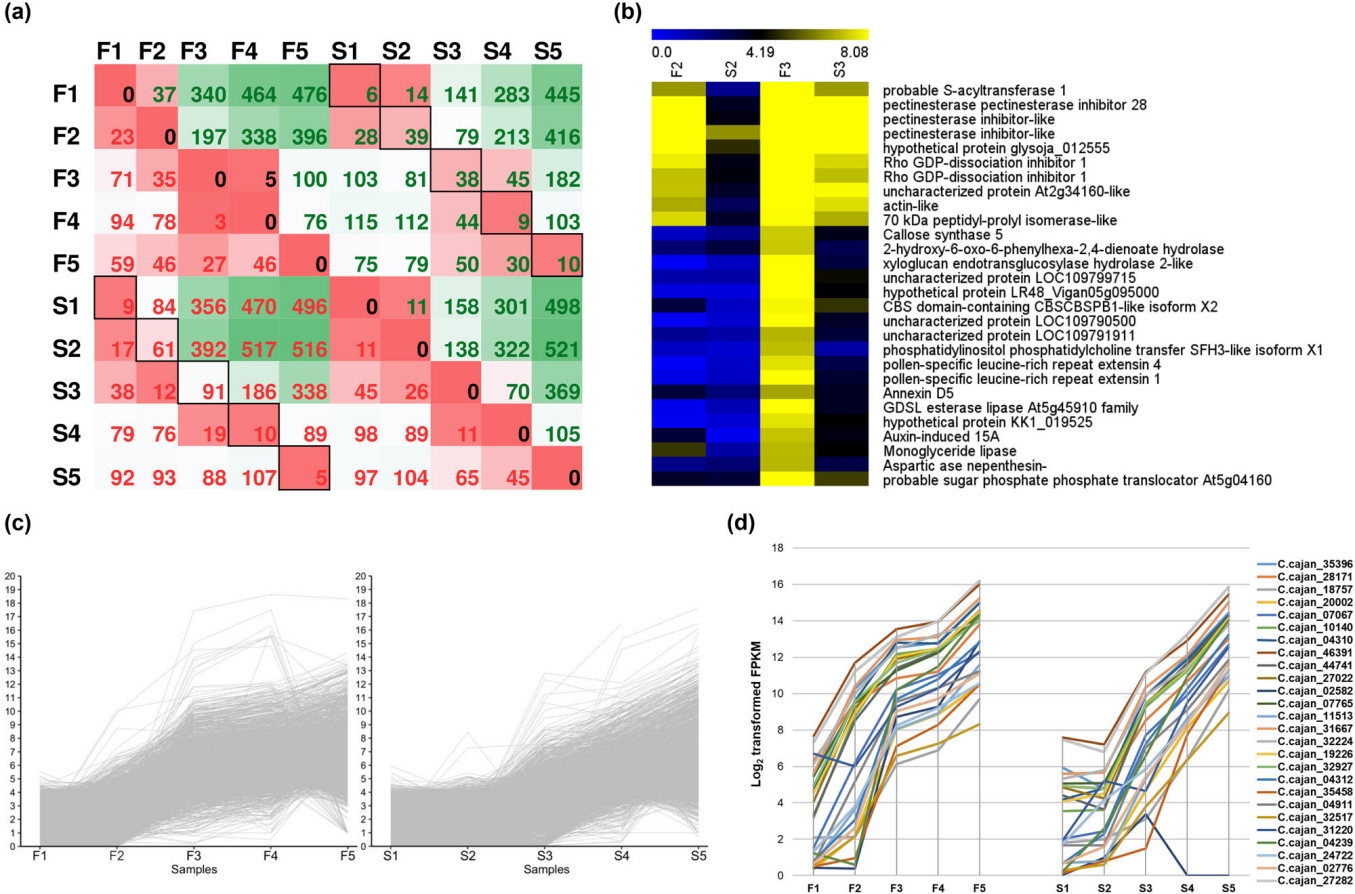
Differentially expressed genes identified among the ten samples. (a) The matrix representing DEGs identified between the ten samples, where each cell in the matrix represents the number of DEGs identified between two samples. The green and the red color of the numbers show the number of identified induced and the repressed genes, respectively. Cells in the matrix have been conditionally formatted with green colour indicating maximum values and red indicating minimum values. (b) Heatmap depicting expression of DEGs generated using the log transformed FPKM during the tetrad and microspore stages in sterile and fertile samples with their corresponding sequence description. The blue colour depicts lower expression and yellow represents higher expression of genes. (c) A co‐expressed gene cluster has been identified in ten anther samples using K‐means clustering, where the first five samples are F1 to F5 followed by S1 to S5. (d) Expression (FPKM values) of a set of selected 23 genes associated with pollen fertility was studied in the five pollen developmental stages. Stages S2 and S3, showed the most substantial changes with majority of genes found repressed in the sterile samples, unlike fertile samples

To investigate the molecular mechanisms and genes involved in fertility transition, sterile and the fertile anther transcriptome profiles were generated using Illumina HiSeq 2500. In total, RNA‐Seq data were generated from sterile (S1, S2, S3, S4 and S5) and fertile (F1, F2, F3, F4 and F5) samples representing five stages of pollen development (Figure 2b1 to 2b6; Supplemental Table S3). In total, 620.77 million reads were generated from 10 libraries that included 337.93 and 282.84 million reads from sterile and fertile samples, respectively. After quality checks and filtering, 313.33 and 258.36 million reads from sterile and fertile samples, respectively were finally processed for downstream analysis which includes mapping, assembly and identification of differentially expressed genes (Supplemental Table S3). About ∼94% reads from each sample could be mapped to the pigeonpea reference genome (Asha V1.0; Varshney et al., 2012), resulting in the identification of 25,230 gene models (Supplemental Table S4), hereafter referred to as genes. Expression of each gene was estimated using fragments per kilo‐base per million reads (FPKM values). The identified genes included 2,330 differentially (Supplemental Table S5), 759 constitutively (Supplemental Table S6) and 126 specifically expressed genes (Supplemental Table S7). Among the non‐redundant set of 2,330 differentially expressed genes (DEGs), 255 DEGs were identified with more than two‐fold differential expression between the sterile and the fertile samples from each of the developmental stages boxed in Figure 3a (Supplemental Table S8).

#### 3.5.1 Tetrad and microspore development stages are critical for fertility transition

Differentially expressed genes (DEGs) were identified in sterile compared to the fertile anther samples and abs (fold change) ≥2 was considered for analysis in all the five developmental stages. Tetrad formation (Stage 2) and microspore development (Stage 3) had the highest number of DEGs identified with 100 and 129 DEGs, respectively, in comparison to 48 DEGs from all the other three Stages 1, 4 and 5 put together (Figure 3a). The number of DEGs identified implies that there is a major difference in the gene expression between sterile and fertile anther transcriptome during these two stages. The heat map (Figure 3b) and the hierarchical clustering (Supplemental Figure S3) of the DEGs clearly indicate two sets of genes that were highly differentially expressed between the sterile and the fertile samples during Stage 2 and Stage 3. Further, a set of 1961 genes has also been identified to be co‐expressed encoding MALE DISCOVERER 2‐like, callose synthase 5, auxin‐induced 15A, pollen‐specific leucine‐rich repeat extension, probable sugar phosphate phosphate translocator At5g04160, and xyloglucan endotransglucosylase hydrolase 2‐like among others (Figure 3c).

Furthermore, a set of 23 genes reported to be associated with pollen fertility in pigeonpea (Pazhamala et al., 2017) was studied in the five developmental stages of fertile and sterile anther samples. All these genes showed highly repressed expression during Stage 2 and Stage 3 in the case of sterile compared to the fertile samples. In all other three stages namely Stage 1, Stage 4 and Stage 5, these genes showed almost similar expression in both sterile and fertile samples (Figure 3d; Supplementa1 Table S9). A pollen‐specific gene encoding an extension‐like domain; *Pex1* (XLOC_021847) showed highly repressed expression throughout the pollen development stage in sterile anthers which could be explained by the fact that this protein *Pex1* typically localized to the intine of the mature pollen (Rubinstein, Broadwater, Lowrey, & Bedinger, 1995) is practically not formed in the case of sterile samples. Higher expression of these genes in fertile compared to sterile samples suggested their critical role in fertility transition at tetrad formation (Stage 2) and microspore development (Stage 3) which corroborates with the cytological analyses revealing an aberration in the post‐meiotic pollen development.

Therefore, in the subsequent analysis, we focussed on the genes that were more than two‐fold differentially induced/repressed between the sterile and the fertile samples in the Stage 2 and Stage 3. We further investigated the genes that were significantly repressed in sterile samples compared to the fertile during these two stages of pollen development in response to moderately elevated day temperature.

#### 3.5.2 Anomaly associated with cell wall during tetrad formation

During the tetrad formation stage, 100 DEGs were identified including 39 induced and 61 repressed in sterile with respect to the fertile samples (Supplemental Table S10). In sterile sample (S2), DEGs were identified encoding proteins associated with cell wall biogenesis, cell wall remodelling, membrane components and membrane transport. Proteins/enzymes including pectinesterase/pectinesterase inhibitor 28 (XLOC_006729), pectinesterase inhibitor‐like (XLOC_036216; XLOC_036217), actin‐like (XLOC_019457), polygalacturonase (XLOC_028604), probable S‐acyltransferase 1 (XLOC_014540), probable UDP‐arabinopyranose mutase 4 (XLOC_050678) and cell differentiation RCD1 homolog (XLOC_004394) showed highly repressed expression. Polygalacturonase has been implicated in pollen mother cell wall degradation for microspore separation (Rhee, Osborne, Poindexter, & Somerville, 2003). On the other hand, certain cell wall modification enzyme associated with saccharification and lignification of the cell wall (Sumiyoshi et al., 2013) such as beta‐xylosidase/alpha‐L‐arabinosidase (XLOC_018096) and cellulose synthase A catalytic subunit 4 (XLOC_025995) were found to be highly up‐regulated in sterile samples, possibly in response to the moderately elevated temperatures in our study. In addition, certain stress‐associated enzymes/proteins including 2, 4‐D inducible glutathione S‐transferase (XLOC_014146), Glutathione S‐transferase (XLOC_030482), kDa class II heat shock (XLOC_036446), heat shock 70 kDa‐like (XLOC_052394), class I heat shock (XLOC_026252; class I heat shock‐like (XLOC_016438; XLOC_026253) were found to be 3.9 to 5.3‐fold repressed than in the case of fertile samples. It is important to mention that 2, 4‐D inducible glutathione S‐transferase has been reported to be auxin induced protein (Flury, Adam, & Kreuz, 1995). Heat shock proteins (Hsp) have been suggested to function as molecular chaperones involved in folding/unfolding of proteins during meiosis and tetrad formation stages (Bukau, Weissman, & Horwich, 2006). Interestingly in sterile samples, two transcription factors namely REVEILLE 1 and zinc finger CONSTANS‐LIKE 12‐like isoform X1 were found to be over 5‐fold repressed. REVEILLE 1 is a morning‐phase transcription factor (TF) that integrates the circadian clock and auxin pathways by regulating auxin level in a time‐of‐day specific manner with no effect during the night (Rawat et al., 2009). A zinc finger CONSTANS‐LIKE 12‐like isoform X1 has also been reported to be a putative TF expressed with a circadian rhythm that peaks during the morning hours (UniProt accession number: O50055). However, these TF were highly induced in the fertile anther samples. In sterile anthers, highly repressed genes (3.2 to 4.1 fold) involved in small GTPase mediated signal transduction including Rho GDP‐dissociation inhibitor 1 (XLOC_025101; XLOC_015903), Rac‐like GTP‐binding RHO1 (XLOC_048195), Rho GTPase‐activating REN1‐like isoform X3 (XLOC_006140), ARF guanine‐nucleotide exchange factor GNL2 (XLOC_043719), CRIB domain‐containing RIC6‐like (XLOC_005006) indicates their role in normal pollen development. In brief, during tetrad formation stage of the sterile anthers, the downregulation of genes encoding cell‐modifying enzymes, stress‐responsive proteins and important transcription factors clearly indicated anomaly in the normal pollen development.

#### 3.5.3 Cell wall plasticity and calcium homeostasis critical during microspore development

In microspore stage (Stage 3), 129 DEGs were identified which include 38 induced and 91 repressed genes (Supplemental Table S11). Since the aberration in pollen development is already indicated from the Stage 2, it would be expected that the effect of the repression of the cellular responses will be perpetuated henceforth. Similar to the Stage 2, genes encoding enzymes regulating cell wall organization/modification/plasticity such as pollen‐specific leucine‐rich repeat extensin 4 (XLOC_014782), callose synthase 5 (XLOC_010646), pectinesterase inhibitor‐like (XLOC_036217), pectinesterase pectinesterase inhibitor 28 (XLOC_006729), ALTERED XYLOGLUCAN 4‐like (XLOC_012492), pollen‐specific leucine‐rich repeat extensin 4 (XLOC_014782), carbon catabolite repressor 4 homolog 3‐like isoform X3 (XLOC_015300) and glycerophosphoryl diester phosphodiesterase 3 (XLOC_039578) were highly repressed. On the other hand, endo‐1,4‐beta‐xylanase A‐like (XLOC_006292) and pectin acetylesterase 9‐like isoform X1 (XLOC_003293) was found to be highly induced in the sterile sample. In addition to these, other proteins such as probable trehalose‐phosphate phosphatase F (XLOC_023629), low‐temperature‐induced 65 kDa‐like (XLOC_019440), auxin‐induced 15A (XLOC_015639), and probable sugar phosphate phosphate translocator At5g04160 (XLOC_026150) also showed down‐regulation. Interestingly, genes implicated in regulating calcium homeostasis have been identified such as repressed levels of calcium‐transporting ATPase (XLOC_040533; XLOC_007875), annexin D5 (XLOC_010180) and vacuolar cation proton exchanger 3 (XLOC_045615), whereas induced level of 45 kDa calcium‐binding (XLOC_001804) and calmodulin‐binding transcription activator 3 (XLOC_027659) encoding gene. An inappropriately located calcium concentration may affect pollen development and seem to have a close correlation to male sterility (Tian, Kuang, Musgrave, & Russell, 1998).

#### 3.5.4 Specifically expressed genes in sterile and fertile anthers

Specifically expressed genes represent the group of genes that have shown expression exclusive to one developmental stage (Supplemental Table S7). A maximum of 41 sample‐specific genes was identified in sterile anthers during desiccated pollen stage at anthesis (Stage 5) and the least number of seven genes during the microspore stage (Stage 3). Genes encoding auxin efflux carrier component 1c in tetrad stage and pectinesterase inhibitor 28 in microspore stage were found exclusively expressed in sterile samples. In the case of the fertile samples, auxin‐induced X10A, benzyl alcohol O‐benzoyltransferase‐like, V‐ATPase‐related vacuolar proton pump‐related, and glutaredoxin encoding genes were identified during the tetrad formation stage. On the other hand, genes encoding proteins such as lignin‐forming anionic peroxidase‐like, auxin‐induced X15 and WUSCHEL‐related homeobox 3 were exclusively identified during fertile microspore stage. Exclusively expressed genes allowed the identification of distinct cellular activities taking place in the sterile and the fertile state of the anthers. Interestingly, auxin‐induced genes in fertile and cell wall modifying enzymes in the case of sterile samples were prominent. This also provided us with the lead to study the role of auxin in reversing male sterility in the TGMS line.

### 3.6 Proteome profiling confirms altered cell wall modification in sterile pollen

In order to complement our comprehensive transcriptomics study, we conducted a systematic proteomic survey on the same set of sterile and fertile anther samples. The sterile and fertile anthers from the five pollen developmental stages led to the identification of 6,652 proteins (Supplemental Table S12), of which 778 non‐redundant proteins were identified as Differentially Expressed Proteins (DEPs) in five pollen developing stages among the sterile and fertile anther samples with ≥2 fold change. During the tetrad formation and microspore development stages, 131 and 114 DEPs were identified, respectively. These proteins include beta‐galactosidase 15‐like (XLOC_035411; XLOC_024856), Beta‐galactosidase 13 (XLOC_009070), beta‐galactosidase‐like isoform X1 (XLOC_052345), starch synthase chloroplastic amyloplastic‐like (XLOC_022799), and mitochondrial proteins (XLOC_035674; XLOC_012840) which were found down‐regulated in sterile samples during these two critical stages. Beta‐galactosidases are known to have an important role in cell wall loosening (Dopico et al., 1998), which is more than 2 fold repressed in sterile samples.

#### 3.6.1 Anther transcriptome and proteome analyses

**FIGURE 4:**
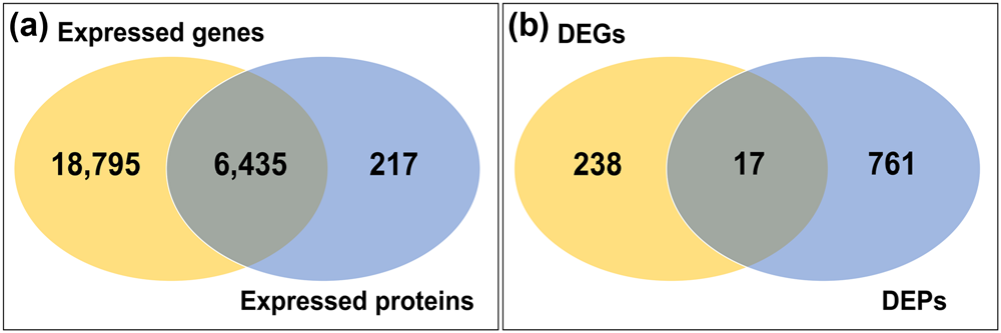
Venn diagram representing the number of genes and proteins identified in sterile and fertile anther samples. (a) Venn diagram represents the number of expressed genes and proteins in sterile and fertile anther samples of different stages. (b) Venn diagram depicts the number of differentially expressed genes and proteins that were identified specifically and commonly using transcriptomic and proteomic approaches

A total of 25,230 genes and 6,652 proteins were identified from the sterile and fertile anthers corresponding to five pollen developmental stages using transcriptomics and proteomics profiling respectively. Out of the total expressed genes and proteins thus identified, 6,435 genes were in common (Figure 4a). Ninety‐six of the proteins identified from the anther proteome data were among the 255 DEGs identified from anther transcriptome. Out of 96 proteins, 41 and 44 proteins from tetrad and microspore development stages, respectively, matched to the DEGs (Supplemental Table S13). DEGs during Stage 2 could be correlated to the encoded proteins from the proteomic profile. Cell wall modifying enzymes such as pectinesterase inhibitor‐like (XLOC_036217), polygalacturonase (XLOC_028604), UDP‐arabinopyranose mutase 4 (XLOC_050678) and beta‐xylosidase/alpha‐L‐arabinosidase (XLOC_018096) were identified using transcriptomics and proteomics. Furthermore, at microspore stage, some other cell wall modifying enzymes including pectin acetylesterase 9‐like isoform X1 (XLOC_003293), pollen‐specific leucine‐rich repeat extensin 4 (XLOC_014782), xyloglucan endotransglucosylase hydrolase 2‐like (XLOC_036073), endo‐1,4‐beta‐xylanase A‐like (XLOC_006292) were also identified. Pollen specific leucine rich repeat extensin is a chimeric protein that contains an LRR domain and an EXT domain (Baumberger et al., 2003), important for normal cell morphogenesis. Proteins such as auxin‐induced 15A (XLOC_015639), probable sugar phosphate translocator At5g04160 (XLOC_026150), Annexin D5 (XLOC_010180), calcium‐transporting ATPase (XLOC_040533; XLOC_007875), calcium‐binding (XLOC_001804) were also identified.

#### 3.6.2 Spatio‐temporal dynamics of anther transcriptome and proteome

Interestingly, among the 255 DEGs and 778 DEPs identified between sterile and fertile anther samples of the five stages, 17 genes/proteins were commonly identified (Figure 4b). These 17 genes/proteins include pollen‐specific leucine‐rich repeat extensin 4 (XLOC_014782), probable UDP‐arabinopyranose mutase 4 (XLOC_050678), probable methyltransferase PMT18 (XLOC_039883), glucan 1,4‐alpha‐glucosidase‐like (XLOC_040293), glycine‐rich cell wall structural 2‐like (XLOC_012261), putative plant invertase/pectin methylesterase inhibitor domain (XLOC_012261) that have an important role in normal cell wall morphogenesis/remodeling were up to 2.8‐fold repressed. DEPs such as delta‐1‐pyrroline‐5‐carboxylate synthase‐like isoform X2 (XLOC_011413), mitochondrial uncoupling 1‐like (XLOC_035674) and 70 kDa peptidyl‐prolyl isomerase‐like (XLOC_039068) showed decreased levels in Stage 3 which are possibly related to tapetal cell development. However, these proteins were identified as encoded by down‐regulated DEG during Stage 2. Other down‐regulated proteins include mitochondrial phosphate carrier mitochondrial‐like (XLOC_048957), Glutathione S‐transferase (XLOC_030482) and Rho GDP‐dissociation inhibitor 1 (XLOC_025101) identified as DEG during Stage 2 were found to be expressed as DEPs during Stage 4. Similarly, GDSL esterase lipase At5g45910 family (XLOC_027967), probable methyltransferase PMT18 (XLOC_039883), Annexin D5 (XLOC_010180), 21 kDa –like (XLOC_024115) and glycine‐rich cell wall structural 2‐like (XLOC_012261) during Stage 3 as DEGs and Stages 4 as DEPs. However, gene XLOC_040293 having glucan 1,4‐alpha‐glucosidase and starch binding activity were identified during Stage 4 in both the transcriptome and proteome datasets (Supplemental Table S14). These candidate genes/proteins have been implicated to be playing an important role in the fertility transition in the TGMS line under study.

### 3.7 Scanning Electron Microscopy and light microscopy reveals sterile and fertile pollen morphology

**FIGURE 5:**
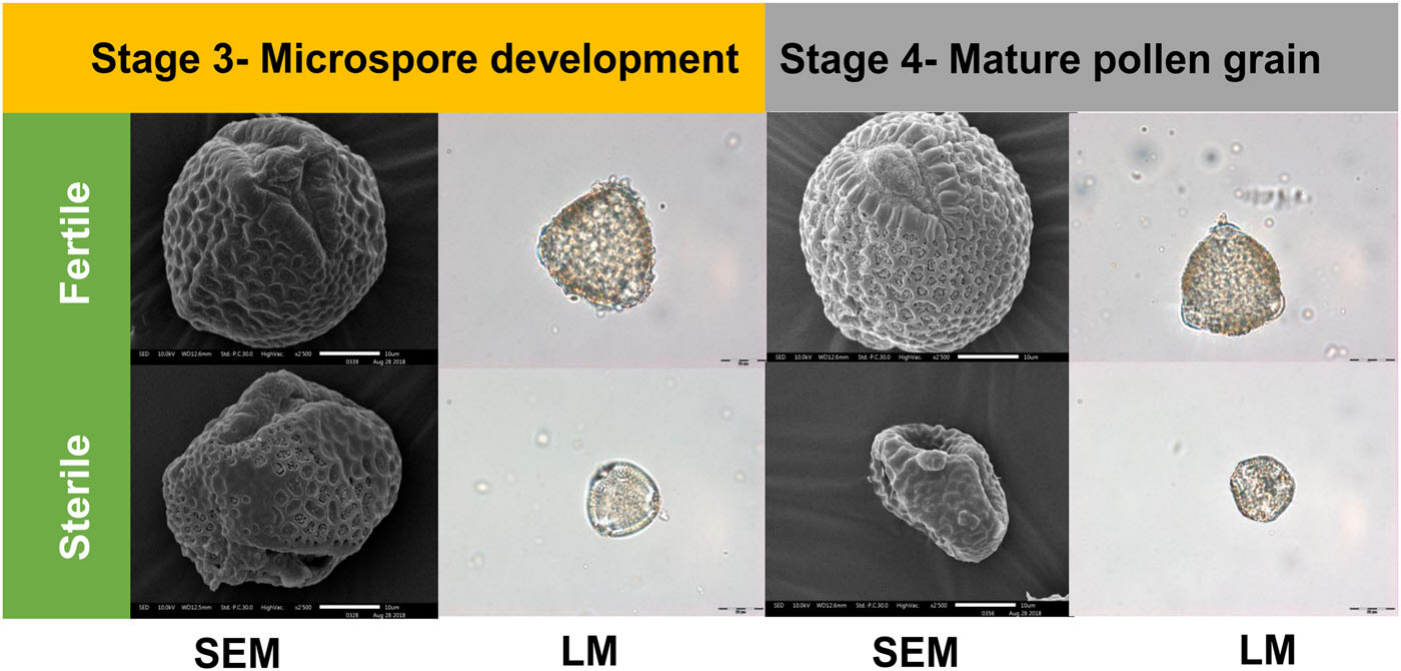
Pollen wall structure using scanning electron microscopy (SEM) and light microscopy (LM). The pollen structure of Stage 3 and Stage 4 from the male sterile and fertile anthers was examined using scanning electron microscopy. The developing microspore and mature pollen grains from the sterile anther samples showed aberration and irregular pollen morphology indicating an altered cell wall modification, unlike the fertile pollen grains

Transcriptome and proteome analysis provided us with the role of various enzymes involved in cell wall organization/modification/plasticity during the tetrad stage (Stage 2) and microspore development (Stage 3). In order to determine the phenotypic changes in pollen due to the down regulation of cell modification protein, we conducted a detailed analysis of the pollen wall in microspore stage and mature pollen (Stage 4) using light microscopy and scanning electron microscopy (SEM). Pollen grains were found to be significantly smaller and irregularly shaped in sterile anthers compared to fertile anthers (Figure 5). Comparison of Stage 3 to Stage 4 showed a shrunken and starved morphological phenotype of the sterile pollen with collapsed apertures. Analysis via light microscopy also revealed that the sterile pollen are smaller, do not contain cell plasma and showed an overall different morphology compared to the fertile pollen grains.

### 3.8 Metabolite profiling reveals impaired sugar utilization in sterile anthers

**FIGURE 6:**
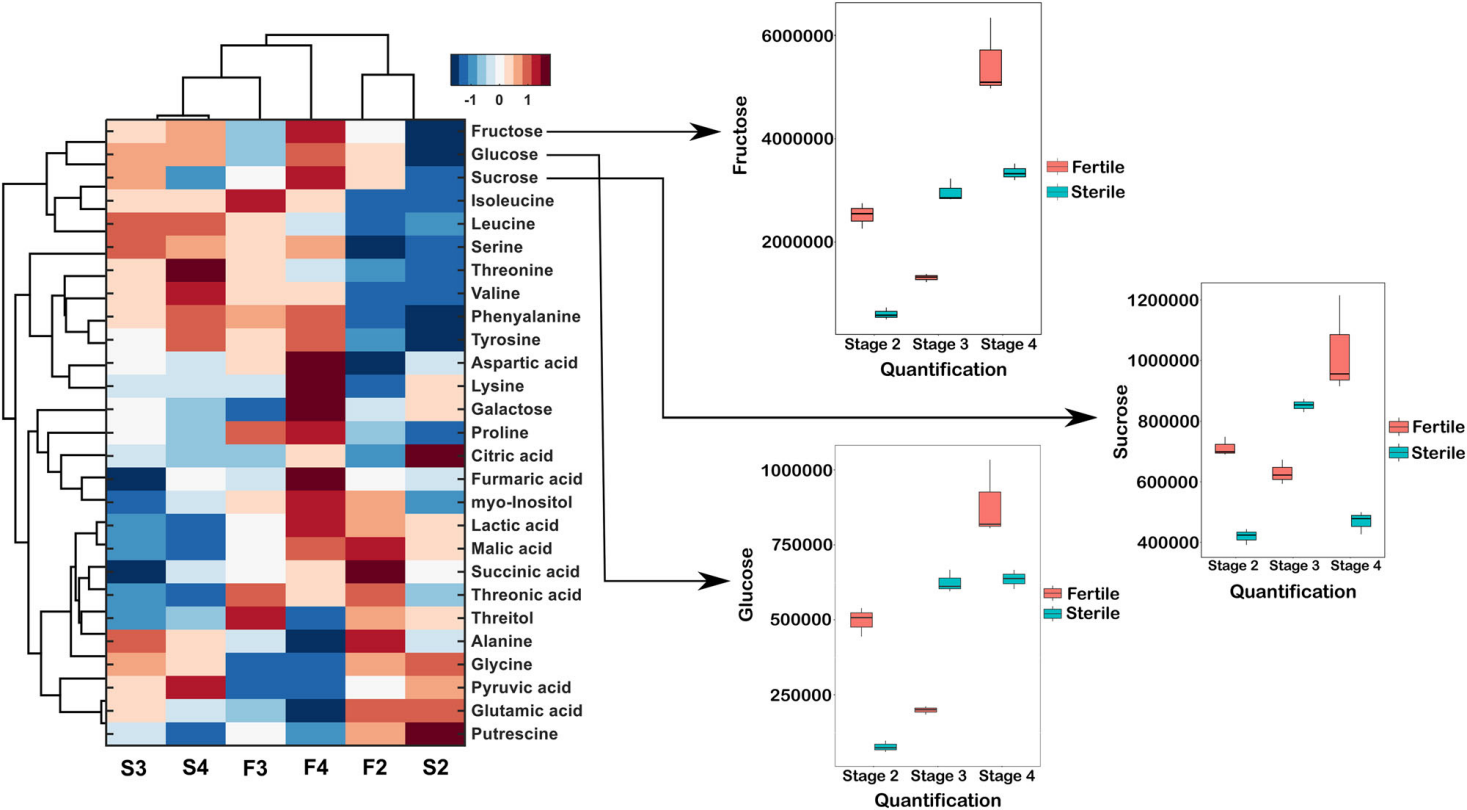
Quantification of primary metabolites in the sterile and fertile anthers during tetrad formation, microspore and mature pollen development. Heatmap shows identification of 27 metabolites from the sterile and fertile anthers of tetrad formation (Stage 2), microspore development (Stage 3) and mature pollen (Stage 4) with box plots highlighting the abundance of sucrose, fructose and glucose in the respective anther stages

Transcriptomic and proteomic analysis identified the down regulation of genes associated with sugar transport such as sugar phosphate translocator At5g04160 (XLOC_026150), sugar transport 10‐like (XLOC_031663), sugar transport 13 (XLOC_011901), sugar carrier C‐like (XLOC_030844), probable galactinol‐sucrose galactosyltransferase 1 (XLOC_002577), glucose‐6‐phosphate 1‐chloroplastic (XLOC_042876) and plastidic glucose transporter 4‐like (XLOC_030669) proteins, especially during Stage 4 and Stage 5 in the microspore stage (Stage 3). To substantiate this finding, we evaluated the abundance of sugars in sterile and fertile anther samples from tetrad, microspore and mature pollen stages through metabolite analysis using GC × GC TOF‐MS. In addition to fructose, glucose and sucrose, 24 other primary metabolites were also identified which could be associated to male sterility. In total, 27 metabolites were explicitly identified in tetrad, microspore and mature pollen stages designated as Stage 2, Stage 3 and Stage 4, respectively (Figure 6). It was observed that Stage 2 of fertile and sterile pollen were clustered together and separated from Stage 3 and Stage 4 of fertile and sterile pollen. This also corroborates with the transcriptomics and proteomics data which demonstrated that the strongest molecular transitions were taking place between tetrads to microspore development. The microspore stage of the sterile samples showed enhanced sugar levels (fructose, glucose, and sucrose) compared to fertile samples (Figure 6).

Conversely, in the other two stages (Stage 2 and Stage 4) in sterile samples, sugar levels were lower in comparison to fertile samples. This accumulation/enhanced levels of sugars in the Stage 3 could possibly be the unutilized sugars indicating an impaired transport during microspore development which correlates with the observed starved morphological phenotypes of the sterile pollen (Figure 2b, 4 to 2b6). Further, metabolites such as myo‐Inositol and threonic acid have been observed to have lower levels in sterile compared to fertile anther samples. This also indicates their important roles in viable pollen formation.

### 3.9 Auxin plays an important role in fertility transition

#### 3.9.1 Pollen viability in response to auxin treatment

Pollen viability analysis carried out on the buds with auxin‐treatment (10^−4^ M IAA solution) and mock‐treatment (sprayed with distilled water) showed clear differences in PSP. Before the treatment, plants were maintained under growth chamber conditions favouring male sterility (13 h day length, 30 °C). The exogenous application of auxin from bud initiation stage to the buds in the tetrad stage of the complete male sterile plants showed the development of buds with viable pollen grains (about 50% PSP). Mock‐treated buds, on the other hand showed more than 90% sterile pollen grains (Supplemental Figure S4a). Auxin treated anthers developed fertile pollen grains in response to the exogenous application of auxin, whereas the untreated anthers remained sterile with non‐viable pollen. This experiment demonstrated that auxin plays a crucial role in promoting male fertility in the male sterile line studied.

#### 3.9.2 Expression analysis of auxin related genes

Auxin related genes identified from the anther transcriptome of Stage 2, Stage 3 and Stage 4 were selected for expression studies. Expression of nine auxin related genes found significantly expressed in the anther transcriptome was analysed in the anther samples that were treated with auxin in relation to the untreated anthers using qPCR (Supplemental Figure S4b). The nine genes considered for this study encoded proteins namely YUCCA6, auxin induced protein ARG7, auxin induced protein 5NG4, auxin induced protein 22D, auxin response factor 8, auxin polar transport, auxin transport BIG. It was found that auxin induced protein ARG7, auxin induced protein 5NG4, and auxin response factor 8 showed an up regulation in the auxin‐treated samples during Stage 2 and Stage 3. On the other hand, all the auxin transport proteins, namely auxin polar transport and auxin transport BIG (XLOC_000496) showed induced expression during Stage 3 and Stage 4. The expression analysis clearly indicated that following exogenous supplementation of the auxin, the expression of auxin induced proteins and auxin transport proteins were able to provide auxin to elicit the downstream signalling for the required cell wall modification so as to be able to obtain nutrients and sugars for normal development of pollen grains. This experiment reveals that there is a crucial decrease in auxin levels that lead to male sterility in the *Envs Sel 107* pigeonpea line.

## 4 DISCUSSION

**FIGURE 7:**
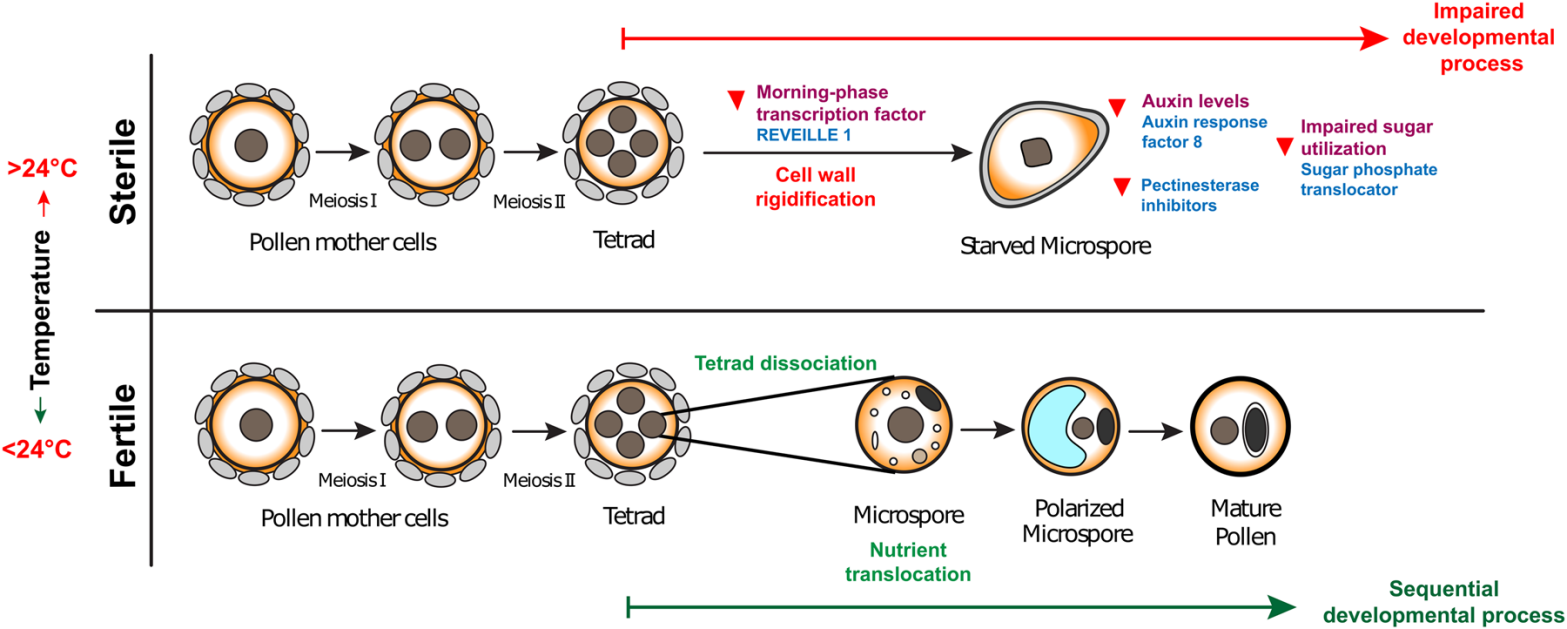
Schematic representation of the molecular mechanism explaining male fertility transition in response to day temperature. The molecular mechanism has been postulated by integrating findings from the phenotypic, cytological and integrated omics studies. Under moderately elevated day temperature, there would be a discrepancy in auxin homeostasis that leads to altered cell modification and subsequent impairment of sugar transport leading to microspore starvation in the male sterile line. On the other hand, under low day temperature, a morning‐phase transcription factor seems to take care of auxin homeostasis and circadian rhythms enabling normal pollen development favouring male fertility

Transcriptomics, proteomics and metabolomics studies along with critical information from the cytological studies of the sterile and the fertile anthers unravelled the anomalies in the cellular and biological processes leading to male sterility (Figure 7). It was observed that male sterile condition could be reversed by reducing the day temperature below the critical threshold temperature of 24 °C. The anthers collected from the pigeonpea plants grown under both permissive (below 23 °C) and restrictive (above 24 °C) temperature conditions were used for comparative multiomics analysis to explore the fertility alteration mechanism.

From our study, transcriptome and proteome analysis of the anthers revealed an interplay of several internal regulators such as light, phytohormones (auxin) that alters downstream signalling (possibly GTPases‐mediated) leading to dynamic changes in the cell wall morphogenesis (biogenesis/organization/modification). The molecular mechanisms by which the environmental cues are perceived and translated into cellular responses to maintain the integrity of the carbohydrate‐rich cell wall for cell growth is less understood (Hord et al., 2008), especially during pollen/anther development.

Our findings from the multiomics data and the cytological data indicate that the transition between male sterility and fertility in the *Envs Sel 107* line takes place during the tetrad and microspore stage of pollen development. Tetrad formation plays a very important role in male gametophyte development which is highly sensitive/responsive to external cues such as temperature (Erickson & Markhart, 2002) and the proteins/genes identified during this stage might play a crucial role in the pollen development (Chaturvedi et al., 2013). During this stage, we identified a morning‐phased transcription factor, REVEILLE 1 that has been previously reported to regulate auxin levels in a time‐of‐day specific manner by integrating circadian clock that peaks during morning hours (Rawat et al., 2009). This explains the fertility/sterility conversion in response to day temperature, especially during morning hours. Many auxin‐induced/polar transport proteins have been identified to be repressed in the case of sterile samples which indicates the role of auxin‐related regulation in *Envs Sel 107* line. We demonstrated that exogenous application of auxin could induce the expression of auxin transport proteins in fertile pollen grains using qPCR analysis. It seems likely that the polar auxin transport in the anther filaments and pollen grains might be blocked which also explains the shorter anther filaments in the male sterile line under study (Feng et al., 2006). Further, the repressed levels of pectinesterase inhibitors during tetrad formation could alter the physiochemical properties of the cell wall components causing sterility, which was evident in the scanning electron microscopic images (Figure 5). Pectinesterase inhibitors have been reported to regulate demethylesterification of cell wall pectins (Jolie, Duvetter, Loey, & Hendrickx, 2010), demethylesterified pectins form Ca^2+^ bonds inducing gel formation that rigidifies the cell wall, affecting cell wall composition (Juge, 2006; Rockel, Wolf, Kost, Rausch, & Greiner, 2008). Formation of cell wall boundaries between the tapetal layer and the middle layer or the pollen wall thickening/rigidification inhibits nutrient flow to the growing microspores, possibly leading to its starvation and subsequent male sterility. Metabolite profiling showed accumulated levels of unutilized sugars in the microspore stage indicating an impaired sugar mobilization and subsequent utilization during the pollen developmental process. The development of microspores is tightly coordinated with tapetal layer development and its timely degeneration, which is very critical for normal pollen development (Parish & Li, 2010).

An integrated‐omics approach supported by precise phenotyping and cytological studies has led us to a systems‐level understanding of the fertility transition in a pigeonpea TGMS line, Envs *Sel 107*. Using this approach, we were also able to determine the precise day temperatures which could be utilized for hybrid seed production (sterility condition) and multiplication of the TGMS line (fertility condition). Accordingly, any fluctuation in the environmental condition could be monitored for critical temperatures. We have also demonstrated that exogenous application of auxin would be useful for multiplication of the male sterile line under unfavourable conditions (e.g. higher day temperatures). In the tropical regions, thermosensitive genic male sterility is considered more appropriate for two‐line hybrid breeding over photo‐sensitive genic male sterility as photoperiod differences are marginal (Virmani, 1996). Thus, the TGMS line identified in this study could be used for developing a two‐line hybrid system in pigeonpea for the semi‐arid tropics.

## Supporting information

SUPPORITNG MATERIAL

## Data Availability

The dataset generated during the current study including the RNA‐Seq data are available at NCBI Sequence Read Archive (SRA) database with BioProject ID #PRJNAH37382.
