# Supplementary material for: Multiomics approach unravels fertility transition in a pigeonpea line for a two‐line hybrid system
Source: Plant Genome. 2020 Jun 18;13(2):e20028. doi: 10.1002/tpg2.20028 (PMC12807119; doi:10.1002/tpg2.20028)
Supplement: Supplementary file 1 [file TPG2-13-e20028-s001.docx]

**Multi-omics approach unravels fertility transition in a pigeonpea line for a two-line hybrid system**

Lekha T Pazhamala^1^, Palak Chaturvedi^2^, Prasad Bajaj^1^, Sandhya Srikanth^1^, Arindam Ghatak^2^, Annapurna Chitikineni^1^, Anke Bellaire^2,3^, Anupama Hingane^4^, CV Sameer Kumar^4^, KB Saxena^4^, Wolfram Weckwerth^2,5*^, Rachit K Saxena^1*^ and Rajeev K Varshney^1,6,*^

^1^Center of Excellence in Genomics & Systems Biology, International Crops Research Institute for the Semi-Arid Tropics (ICRISAT), Patancheru - 502 324, Hyderabad, India

^2^Molecular Systems Biology (MOSYS), Department of Functional and Evolutionary Ecology, Faculty of Sciences, University of Vienna, Althanstrasse 14, 1090, Vienna, Austria

^3^Department of Botany and Biodiversity Research, University of Vienna, Rennweg 14, 1030, Vienna, Austria

^4^Crop Improvement Theme, International Crops Research Institute for the Semi-Arid Tropics (ICRISAT), Patancheru - 502 324, Hyderabad, India

^5^Vienna Metabolomics Center (VIME), University of Vienna, Vienna, Austria

^6^Institute of Agriculture, University of Western Australia, 35 Stirling Highway, Crawley, WA, 6009, Australia

^*^Correspondence:

**Rajeev K Varshney** and **Rachit K Saxena**, Center of Excellence in Genomics & Systems Biology, ICRISAT, Patancheru – 502 324, Hyderabad, India.

Tel.: +91 40 3071 3305; fax: +91 40 3071 3074/ 3075

**Wolfram Weckwerth**, Department of Ecogenomics and Systems Biology, Faculty of Sciences, University of Vienna, Althanstrasse 14, 1090, Vienna, Austria

Tel.: +43-1-4277-577 00

E-mail addresses: [r.k.varshney@cgiar.org](mailto:r.k.varshney@cgiar.org) (Rajeev K Varshney), [r.saxena@cgiar.org](mailto:r.saxena@cgiar.org) (Rachit K Saxena) and [wolfram.weckwerth@univie.ac.at](mailto:wolfram.weckwerth@univie.ac.at) (Wolfram Weckwerth)

**Running title: Multi-*omics* approach unravels fertility transition**

**Supplemental Materials**

**Figure S1. Evaluation of eight EGMS lines for fertility transition under field conditions. a)** Minimum and maximum temperatures recorded during June 2012 to February 2013. **b)** EGMS lines showing fertility transition under field conditions grown in three successive months during 2012. **c)** Evaluation of the three EGMS lines namely *Envs Sel 104*, *Envs Sel 105* and *Envs Sel 107* under lower temperature and short-day conditions. **d)** Evaluation of the three EGMS lines namely *Envs Sel 104*, *Envs Sel 105* and *Envs Sel 107* under higher temperature and long day conditions.

**Figure S2. Sterile and fertile anthers and pollen morphology.**

The top row shows the morphology of sterile anthers and pollen which has been compared to the bottom row showing fertile anthers and pollen of the same developmental stage. **a)** Sterile anthers at the dehiscence stage. **b)** Fertile anthers after dehiscence **c)** Sterile anthers before dehiscence stage. **d)** Fertile anthers before dehiscence (Bar: 2 mm). **e)** Non-viable sterile pollen devoid of stain from sterile anthers. **f)** Brightly stained viable pollen grains from fertile anthers with 1% acetocarmine (Bar: 100µm). **g)** A magnified image of the sterile pollen that remained intact in the tetrad condition from sterile anther. **h)** Fertile pollen stacked within the fertile anther locule.

**­­­**

**Cluster A**

**Cluster B**

**Figure S3. Global analysis of differentially expressed genes between sterile and fertile samples.**

Hierarchical clustering indicates expression of differentially expressed genes (rows) across ten sterile and fertile samples (fold-change > 2, P < 0.05) with their corresponding sequence description. Hierarchical clustering was performed with the log transformed FPKM values using Pearson correlation. Cluster A depicts genes highly induced in F3 while highly repressed in S3 and cluster B represents genes highly induced in F2 while highly repressed in S2. The blue colour depicts lower expression and yellow represents higher expression of genes.

**b**

**Figure S4: Auxin treatment reverses male sterility in Envs Sel 107 line.**

**a)** Effect of auxin on the pollen viability of male sterile Envs Sel 107 line. Exogenous application of IAA showed improvement in the percentage of viable pollen compared to the mock-treated male sterile plants. The photomicrograph of the auxin-treated and mock-treated pollen is in the same scale (100 µm). **b)** Expression analysis of auxin related proteins encoding genes identified from the anther transcriptome data in response to exogenous auxin application with respect to the untreated plants using qPCR.
